# Supplementary material for: Forecasting for Haditha reservoir inflow in the West of Iraq using Support Vector Machine (SVM)
Source: PLoS One. 2024 Sep 6;19(9):e0308266. doi: 10.1371/journal.pone.0308266 (PMC11379161; doi:10.1371/journal.pone.0308266)
Supplement: Attachment — Submitted filename: PONE-D-24-07720 - Response to Reviewers 4.docx [file pone.0308266.s003.docx]

PONE-D-24-07720

Forecasting for Haditha Reservoir Inflow in the West of Iraq Using Support Vector Machine

PLOS ONE

**Editor Comments**

- **Kindly add more chart for better analysis**

Author response: The authors greatly appreciate to the Editor’s comments.

The (Fig.8) shows the difference in the value of MAE between the daily, monthly and seasonal discharges in the testing phase of the models used. It is noted that the lowest value of the statistical coefficient MAE was on the daily discharges of the first model using the linear kernel function, where its value reached (33.26), which is the lowest value compared to the monthly and seasonal discharges. The same applies to the other kernels, which are Gaussian and quadratic. While the (Fig. 9) shows the difference in the value of RMSE between the daily, monthly and seasonal discharges in the testing phase of the models used. It is noted that the lowest value of the statistical coefficient RMSE was on the daily discharges of the first model using the linear kernel function, where its value reached (53.29), which is the lowest value compared to the monthly and seasonal discharges. The same applies to the other kernels, which are Gaussian and quadratic.

AME

Discharges (m3/sec)

| Models | Daily | Monthly | Seasonal |
| --- | --- | --- | --- |
| Linear -Moddel 1 | 33.26 | 76.23 | 131.5 |
| Linear -Moddel 2 | 33.86 | 78.45 | 129.5 |
| Linear -Moddel 3 | 33.66 | 76.08 | 130.4 |
| Linear -Moddel 4 | 33.49 | 76.61 | 140.8 |
| Linear -Moddel 5 | 33.54 | 77.76 | 136.7 |
| Qudratic - Moddel 1 | 42.7 | 76.23 | 129.3 |
| Qudratic - Moddel 2 | 49.3 | 76.17 | 119.87 |
| Qudratic - Moddel 3 | 38.22 | 72.68 | 119.26 |
| Qudratic - Moddel 4 | 39.97 | 72.97 | 159 |
| Qudratic - Moddel 5 | 34.52 | 75.99 | 161.7 |
| Fine -Moddel 1 | 37.7 | 93.82 | 149.8 |
| Fine -Moddel 2 | 39.01 | 109.9 | 166.8 |
| Fine -Moddel 3 | 40.41 | 105.86 | 163.2 |
| Fine -Moddel 4 | 40.78 | 113.2 | 178.3 |
| Fine -Moddel 5 | 41.04 | 119.46 | 196.2 |
| Mid - Moddel 1 | 35.05 | 80.43 | 136.7 |
| Mid - Moddel 2 | 36.51 | 89.19 | 139.2 |
| Mid - Moddel 3 | 35.1 | 89.48 | 143.6 |
| Mid - Moddel 4 | 35.78 | 87.36 | 162.2 |
| Mid - Moddel 5 | 35.78 | 86.25 | 158.1 |
| Coarse - Moddel 1 | 35.61 | 80.43 | 137.7 |
| Coarse - Moddel 2 | 36.94 | 83.06 | 140.3 |
| Coarse - Moddel 3 | 36.52 | 82.16 | 142.2 |
| Coarse - Moddel 4 | 35.64 | 82.74 | 146.1 |
| Coarse - Moddel 5 | 36.37 | 83.61 | 145.3 |

**Fig 8.** **AME value for the testing phase SVM model for daily, monthly, and seasonal discharges**

RMSE

Discharges (m3/sec)

| Models | Daily | Monthly | Seasonal |
| --- | --- | --- | --- |
| Linear -Moddel 1 | 53.29 | 111.3 | 153.6 |
| Linear -Moddel 2 | 54.05 | 110.5 | 154.5 |
| Linear -Moddel 3 | 54.2 | 109.4 | 159 |
| Linear -Moddel 4 | 53.83 | 109.8 | 176.1 |
| Linear -Moddel 5 | 54.01 | 110.2 | 171.5 |
| Qudratic - Moddel 1 | 58.41 | 111.3 | 152.3 |
| Qudratic - Moddel 2 | 64.21 | 113.8 | 160.1 |
| Qudratic - Moddel 3 | 56.65 | 111.9 | 158.75 |
| Qudratic - Moddel 4 | 57.41 | 111.9 | 198.3 |
| Qudratic - Moddel 5 | 55.08 | 115.4 | 202.4 |
| Fine -Moddel 1 | 61.1 | 141.4 | 175.3 |
| Fine -Moddel 2 | 65.5 | 157 | 203.4 |
| Fine -Moddel 3 | 68.8 | 148 | 188.9 |
| Fine -Moddel 4 | 69 | 155 | 203.6 |
| Fine -Moddel 5 | 68.7 | 159 | 227 |
| Mid - Moddel 1 | 53.45 | 112.5 | 158.8 |
| Mid - Moddel 2 | 54.79 | 119.4 | 170.6 |
| Mid - Moddel 3 | 54.79 | 118.6 | 174.1 |
| Mid - Moddel 4 | 54.73 | 118.3 | 195.2 |
| Mid - Moddel 5 | 54.89 | 115.9 | 192.1 |
| Coarse - Moddel 1 | 53.67 | 112.45 | 158.7 |
| Coarse - Moddel 2 | 54.87 | 112.17 | 162.6 |
| Coarse - Moddel 3 | 54.68 | 112.7 | 166.2 |
| Coarse - Moddel 4 | 53.96 | 113.3 | 176.1 |
| Coarse - Moddel 5 | 54.25 | 114.9 | 171.4 |

**Fig 9.** **RMSE value for the testing phase SVM model for daily, monthly, and seasonal discharges**

- **Compare the result with previous study.**

Author response: The authors greatly appreciate to the Editor’s comments.

Many global researches have used the same model for future prediction as [12-16-23-28] was used on certain regions. In this study, the same model was used to predict the future daily, monthly, and seasonal discharges of the Euphrates River upstream of the Haditha Dam reservoir. After analyzing the results, we find that the model used has an acceptable performance in future prediction, and compared to the results of other research, there are no significant differences between the results. From observing the results obtained from applying the (SVM) method on the Euphrates River on a daily basis and comparing them with previous studies such as [29][30][31] we notice that he values of statistical coefficients such as (MAE) were close or close to the general average of these studies, as well as the values of (RMSE) and (R2) When applied on a monthly or seasonal basis, the results were acceptable, but not as accurate as the daily forecast.

All comments were responded to and implemented
