# Supplementary material for: Forecasting for Haditha reservoir inflow in the West of Iraq using Support Vector Machine (SVM)
Source: PLoS One. 2024 Sep 6;19(9):e0308266. doi: 10.1371/journal.pone.0308266 (PMC11379161; doi:10.1371/journal.pone.0308266)
Supplement: Attachment — Submitted filename: PONE-D-24-07720 - Response to Reviewers 5.docx [file pone.0308266.s004.docx]

PONE-D-24-07720

Forecasting for Haditha Reservoir Inflow in the West of Iraq Using Support Vector Machine

PLOS ONE

**Editor Comments**

- **Kindly add more chart for better analysis**

Author response: The authors greatly appreciate to the Editor’s comments.

1- All citations mentioned in the manuscript belong to my work and are part of the research methodology.
2- Regarding Figure (1), the issue related to copyright was solved by replacing the image of the study site with that of the source for Figure (1) and as shown in the manuscript in the Study Area section.

**A**

**B**

**C**

**Figs 4. Training process of the SVM model for discharges (a) daily , (b) monthly, and (c) seasonal.**

**R^2^ = 0.96**

**A**

**R^2^ = 0.21**

**C**

**B**

**R^2^ = 0.68**

**Fig 5. R^2^-value of observed versus predicted flow for the training phase SVM model for discharges (a) daily, (b) monthly, and (c) seasonal**

**Fig. 1 The red circle indicates the Location of the study area, the Haditha Dam reservoir location in Iraq** [21]**.**

All comments were responded to and implemented
